# 86583 The role of creatine in developmental myelination and remyelination

**DOI:** 10.1017/cts.2021.655

**Published:** 2021-03-30

**Authors:** Lauren Rosko, Tyler Gentile, Victoria Smith, Jeffrey K. Huang

**Affiliations:** 1 Georgetown University; 2 George Washington University

## Abstract

ABSTRACT IMPACT: This study highlights the importance of creatine in developmental myelination and remyelination to investigate whether creatine provides a therapeutic value during a central nervous system (CNS) demyelinating insult with a potential value in patients with Multiple Sclerosis. OBJECTIVES/GOALS: Creatine is vital for ATP buffering in the brain. Interestingly, the cells that generate myelin express the main enzyme for creatine synthesis, Gamt. Patients with Gamt mutations display intellectual delays and impaired myelination. Therefore, we hypothesize that creatine is essential for developmental myelination and improves remyelination. METHODS/STUDY POPULATION: To investigate these hypotheses, we developed a new transgenic mouse model with LoxP sites flanking exons 2-6 of the guanidinoacetate methyltransferase (Gamt) gene where excision leads to expression of a green fluorescent tag allowing us to track the cells normally expressing Gamt. We used immunohistochemistry techniques to look at the corpus callosum, the main white matter tract in the brain, and evaluate the number of oligodendrocytes (OL), glial cells responsible for generating myelin. We also used the cuprizone model of toxic demyelination to investigate whether dietary creatine and cyclocreatine, a planar analog of creatine that more efficiently crosses the blood-brain barrier, can enhance remyelination. RESULTS/ANTICIPATED RESULTS: In this mouse model, we show a 95% (+/-0.47%, n=3) co-localization of Gamt within mature OL during postnatal (P) day P14 with no co-localization in neurons or other glial cells. This suggests that mature OL are the main cells making creatine in the CNS. Next, we show that knocking out Gamt leads to a significant reduction in OL in the developing corpus callosum, at P14 and P21 (P14: 0.007, n=3; P21: 0.04, n=3). We also show that creatine supplementation causes a trending increase in mature OL density in the corpus callosum following cuprizone demyelination (2% creatine; p=0.052; n=4). Interestingly, cyclocreatine supplementation significantly increased mature OL density in the corpus callosum following cuprizone demyelination (0.1% cyclocreatine; p=0.034; n=4). DISCUSSION/SIGNIFICANCE OF FINDINGS: These studies highlight the important role creatine plays in developmental myelination and remyelination to investigate whether creatine and cyclocreatine provide a therapeutic value during a CNS demyelinating insult. This work investigates a potential therapeutic value of creatine to patients with Multiple Sclerosis.

